# Multiclass classification of imagined speech EEG using noise-assisted multivariate empirical mode decomposition and multireceptive field convolutional neural network

**DOI:** 10.3389/fnhum.2023.1186594

**Published:** 2023-08-10

**Authors:** Hyeong-jun Park, Boreom Lee

**Affiliations:** ^1^Department of Biomedical Science and Engineering, Gwangju Institute of Science and Technology, Gwangju, Republic of Korea; ^2^AI Graduate School, Gwangju Institute of Science and Technology, Gwangju, Republic of Korea

**Keywords:** brain-computer interfaces, imagined speech EEG, multiclass classification, multireceptive field convolutional neural network, noise-assisted empirical mode decomposition

## Abstract

**Introduction:**

In this study, we classified electroencephalography (EEG) data of imagined speech using signal decomposition and multireceptive convolutional neural network. The imagined speech EEG with five vowels /a/, /e/, /i/, /o/, and /u/, and mute (rest) sounds were obtained from ten study participants.

**Materials and methods:**

First, two different signal decomposition methods were applied for comparison: noise-assisted multivariate empirical mode decomposition and wavelet packet decomposition. Six statistical features were calculated from the decomposed eight sub-frequency bands EEG. Next, all features obtained from each channel of the trial were vectorized and used as the input vector of classifiers. Lastly, EEG was classified using multireceptive field convolutional neural network and several other classifiers for comparison.

**Results:**

We achieved an average classification rate of 73.09 and up to 80.41% in a multiclass (six classes) setup (Chance: 16.67%). In comparison with various other classifiers, significant improvements for other classifiers were achieved (*p*-value < 0.05). From the frequency sub-band analysis, high-frequency band regions and the lowest-frequency band region contain more information about imagined vowel EEG data. The misclassification and classification rate of each vowel imaginary EEG was analyzed through a confusion matrix.

**Discussion:**

Imagined speech EEG can be classified successfully using the proposed signal decomposition method and a convolutional neural network. The proposed classification method for imagined speech EEG can contribute to developing a practical imagined speech-based brain-computer interfaces system.

## 1. Introduction

Brain-computer interfaces (BCIs) use brain signals to control machines and allow individuals to communicate with the outside world. BCI implementation requires careful selection of specific brain signals to perform particular tasks. Many signal measurement methods, such as electroencephalography (EEG), near-infrared spectroscopy (NIRS), magnetoencephalography (MEG), and electrocorticography (ECoG), are used for measuring various brain signals for BCI implementation (Bojak and Breakspear, 2013; Chaudhary et al., 2016; Bakhshali et al., 2020). EEG measures the brain’s electrical activity through electrodes spaced on the scalp (Bojak and Breakspear, 2013). EEG has many limitations, such as low spatial resolution, non-stationarity, and low signal-to-noise ratio. However, it is used in many BCIs systems because of its high temporal resolution, noninvasiveness, and cost-friendly characteristics; other signals do not provide these advantages (Bojak and Breakspear, 2013; Pandarinathan et al., 2018; Bakhshali et al., 2020).

There are some limitations in BCIs studies. For example, BCIs based on motor imagery (MI) are limited by the maximum allowable number of tasks (Wang et al., 2013; García-Salinas et al., 2019). Visual stimulus-based BCIs, such as P300 and SSVEP, are limited by more extended periods for obtaining results and the necessity of an external device to generate stimuli (Nguyen et al., 2018; Kaongoen et al., 2021). Therefore, many studies have been conducted on imagined speech-based BCIs to solve these problems and act as an alternative BCI system.

An algorithm that extracts features and classifies EEG is one of the most significant parts of BCI systems. Deng et al. (2010) classified imagined syllables /ba/ and /ku/ in three different rhythms using the Hilbert–Huang transform, and their classification accuracy was more significant than the chance level. Dasalla et al. (2009) proposed a method to classify imagined vowels /a/ and /u/, and resting-state using a common spatial pattern (CSP) filter and support vector machine (SVM) in a binary classification manner. Their classification results were up to 82% (Dasalla et al., 2009). D’Zmura et al. (2009) conducted a speech imagery experiment with four study participants to extract features in wavelet envelope in the theta (3–8 Hz), alpha (8–13 Hz), and beta (13–18 Hz) bands. Their highest accuracy was reported at the beta band of speech imagery /ba/ and /ku/ (D’Zmura et al., 2009). Kaongoen et al. (2021) revealed that the gamma (30–100 Hz) band activity has the highest mean *F*-score in ear-EEG and scalp-EEG during imagined words “right,” “left,” “forward,” and “go back”. As such, several studies have reported that different frequency bands are related to imagined speech. Research on feature-based deep learning in BCIs is progressing with the advancements in deep learning.

Common spatial pattern-based deep learning algorithms have also been proposed in MI BCIs (Kumar et al., 2017; Sakhavi et al., 2018; Zhu et al., 2019). Kumar et al. (2017) proposed CSP to extract features fed into a multilayer perceptron (MLP) to classify MI EEG signals. Sakhavi et al. (2018) proposed combining the filter-bank CSP (FBCSP) and Hilbert transform to extract spatial and temporal features that were used as input for five-layer convolutional neural networks (CNNs) for MI EEG classification. Unlike MI BCIs, imagined speech BCI has been widely proposed as a discrete wavelet transform (DWT)-based deep learning technique (Rezazadeh Sereshkeh et al., 2017; Cooney et al., 2020; Pawar and Dhage, 2020; Panachakel and Ramakrishnan, 2021a). Rezazadeh Sereshkeh et al. (2017) used DWT to extract features and a regularized neural network to classify imagined speech “yes” and “no”. Pawar and Dhage (2020) used DWT as a feature extraction method and an extreme learning machine based on a feed-forward neural network to classify imagined words “left,” “right,” “up,” and “down” and achieved a maximum multiclass calssification accuracy of 49.77%. Cooney et al. (2020) used relative wavelet energy features and several CNNs (shallow CNN, deep CNN, and EEGNet) with different hyperparameters to classify two different imagined speech datasets. Panachakel and Ganesan (2021b) used ResNet50 to classify imagined vowels (two classes) and short-long words (three classes) and obtained classification accuracy of 86.28 and 92.8%, respectively. Lee et al. (2020) achieved 13 class (12 words/phrases and rest state) classification accuracy of 39.73% using frequency band spectral features and SVM with RBF kernel classifier. Li et al. (2021) used a hybrid-scale spatial-temporal dilated convolution network for eight imagined Chinese words EEG with 54.31% classification accuracy and compared it with various classification methods, including EEGNet. However, the usage of deep learning is lacking in the multiclass classification of imagined speech EEG compared to MI EEG (Altaheri et al., 2021; Lopez-Bernal et al., 2022).

**FIGURE 1:**
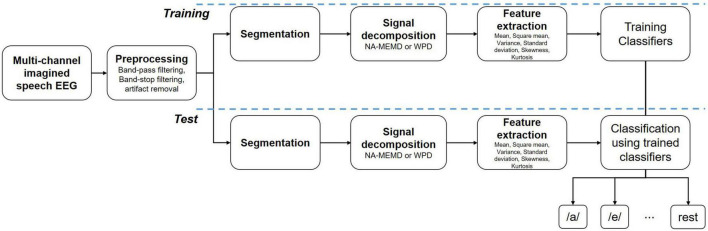
Block diagram for the proposed method.

In this study, we classified five vowel speech imagery and resting-state EEG, as displayed in Figure 1. First, the imagined speech EEG data was preprocessed using a band-pass filter, band-stop filter, and artifact removal via visual rejection. Then, EEG data were divided into training and test datasets using 10 × 10-fold cross-validation. After that, the EEG data were segmented and separated into different frequency bands through noise-assisted multivariate empirical mode decomposition (NA-MEMD). Finally, six statistical features were extracted from the separated frequency bands. The extracted feature vectors were then classified through the proposed multireceptive field CNN (MRF-CNN) and various comparison classifier methods.

Although deep learning has been applied as a state-of-the-art method in many fields of EEG research, such as emotion classification, MI, and covert speech, many other machine learning-based classification methods are still used as much as deep learning. So, several machine learning and end-to-end CNN-based deep learning algorithms and wavelet packet decomposition (WPD), a widely known signal decomposition method, were compared with the proposed deep MRF-CNN with NA-MEMD.

The remaining of this paper is organized as follows. The section “Materials and methods” explains the experimental paradigm, details regarding the participants, EEG preprocessing, NA-MEMD, and the proposed MRF-CNN. Then, the section “Results” compares the classification performance of the proposed architecture and other algorithms. Finally, sections “Discussion” and “Conclusion” have been included at the end of the article.

## 2. Materials and methods

### 2.1. Participants

Nine healthy participants (gender: male, average age: 26.78 ± 2.94 years, range: 24–32 years) were recruited for the study. All participants were native Korean and had no known neurological diseases or other specific health problems. The experimental procedure passed the International Review Board of Gwangju Institute of Science and Technology (No. 20141218-HR-16-01-02). All participants provided written consent for the experimental procedure before the experiments.

### 2.2. Experimental paradigm

e-Prime 2.0 software (Psychology Software Tools, Inc., Sharpsburg, PA, USA) was used to design the experimental procedure. EEG signals were recorded at a sampling rate of 1,000 Hz (Net Station version 4.5.6) using a HydroCel Geodesic Sensor Net with 64 channels and Net Amps 300 amplifiers (Electrical Geodesics, Inc., Eugene, OR, USA). EEG sensors were placed according to the international 10–20 system.

**FIGURE 2:**
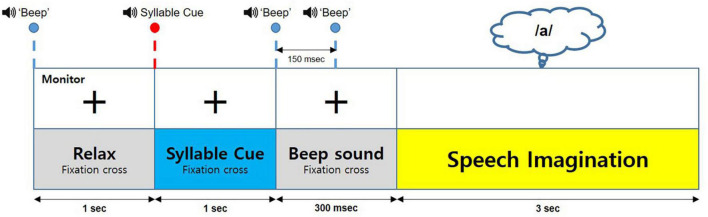
Experimental procedure.

The participants sat in comfortable armchairs at a certain distance from a computer monitor that provided visual stimulation. They wore earphones to listen to the voice stimulation. Five vowel stimuli, namely /a/, /e/, /i/, /o/, and /u/, and a rest (mute sound) stimulus, were used in this experiment. All voice stimuli were recorded using the Goldwave software (GoldWave, Inc., St. John’s, NL, Canada), and source audio was obtained from oddcast’s online. Figure 2 displays the experimental paradigm. Each trial shows a beep and cross mark that denotes a preparation period for the participant before listening to the target vowel. After 1 s, the target vowel stimulus is provided to the participant. Each stimulus was given to the participants randomly. Next, 1 s after the target vowel stimulus, two beeps at intervals of 150 ms are provided to the participant as a preparation for the vowel imagery. The cross mark disappears after the beeps, and the participant is then instructed to imagine the target vowel stimulus for 3 s. Each session repeats each vowel stimulus 10 times, and the participant performs 5 sessions within a day.

### 2.3. EEG processing and NA-MEMD

For EEG preprocessing, we first resampled the acquired EEG data into 250 Hz for the fast preprocessing procedure. Then, the EEG data were high-pass filtered with 0.1 Hz. Next, an IIR notch filter (Butterworth; order: 4; bandwidth: 59–61 Hz) was applied to remove the power line noise. After filtering, to obtain a clean EEG signal, a noisy trial was rejected via Fieldtrip (Oostenveld et al., 2011). Then, using 10 × 10-fold cross-validation, the EEG data were divided into a training set and a test set so that the training set and the test set were not mixed in the subsequent process. Finally, to obtain enough samples for training and testing the classifier, we divided each 3 s trial into five segments with a 1 s duration and 0.5 s overlap. Therefore, the amount of each vowel imagery EEG data was up to 250 if not removed via noisy trial rejection.

Empirical mode decomposition (EMD) is a data-driven single-channel signal decomposition algorithm proposed by Huang et al. (1998) that uses a sifting algorithm to extract intrinsic mode functions (IMFs) from the original signal. Several versions of EMD have been proposed thus far. Ensemble EMD (EEMD) applies EMD after directly mixing noise with a signal (Wu and Huang, 2009). It has a better signal decomposition performance than EMD due to the influence of noise. Multivariate EMD (MEMD) forms a natural extension algorithm of EMD that can be used for multivariate signals because EMD cannot be applied to multivariate signals (Rehman and Mandic, 2010; Ur Rehman et al., 2013; Zhang et al., 2017). NA-MEMD is safer than EEMD for noise effects as it does not directly add noise to the signal. Instead, it adds an extra noise channel with the same length as the existing multivariate signal (Ur Rehman and Mandic, 2011; Ur Rehman et al., 2013). Standard EMD decomposes the time domain signal *y*(*t*) into a finite set of IMFs and residue as follows:


(1) $$y\,\left(t\right)=\sum_{n=1}^{N}D_{n}\,\left(t\right)\,r\,\left(t\right),$$

where *D*_*n*_(*t*) and *r*(*t*) represent the IMFs and residue of the signal, respectively. *D*_*n*_(*t*) is calculated from the local extrema (local maxima and local minima) of the original signal as follows:


(2) $$m\,\left(t\right)=\frac{E_{m\,a\,x}\,\left(t\right)+E_{m\,i\,n}\,\left(t\right)}{2},$$

(3) $$D_{n}\,\left(t\right)=y\,\left(t\right)-m\,\left(t\right),$$

where *E*_*max*_(*t*) and *E*_*min*_(*t*) respectively represent the envelope of local maxima and local minima, and *m*(*t*) represents the mean envelope.

The local mean of an *n*-dimensional signal cannot be calculated directly, as opposed to standard EMD. Therefore, a multidimensional signal envelope is generated from multiple signal projections of an input signal, and the local mean is calculated by averaging the generated envelopes. NA-MEMD applies MEMD after adding an independent white Gaussian noise channel to the multidimensional signal (Ur Rehman et al., 2013). The detailed procedure for the NA-MEMD algorithm is as follows:

1. Generate a *q*-channel uncorrelated white Gaussian noise time series which has the same length as the input signal, with *q* ≥ 1.
2. Add the generated noise channels to the multivariate *n*-channel input signal (*n* ≥ 1) to obtain (*n* + *q*) dimensional multivariate input signal.
3. Choose a suitable point set for sampling on an (*n* + *q* − 1) sphere.
4. Calculate a projection ${\left\{p^{\theta_{k}}\,\left(t\right)\right\}}_{t=1}^{T}$ of the input signal ${\left\{v\,\left(t\right)\right\}}_{t=1}^{T}$ along the direction vector *x*^*θ*_*k*_^ for all *k* (the whole set of direction vectors), which gives ${\left\{p^{\theta_{k}}\,\left(t\right)\right\}}_{k=1}^{K}$ set of projections.
5. Find the time instants $t_{j}^{\theta_{k}}$ corresponding to the maxima of the set of projected signals ${\left\{p^{\theta_{k}}\,\left(t\right)\right\}}_{k=1}^{K}$.
6. Interpolate $\left[t_{j}^{\theta_{k}},v\,\left(t_{j}^{\theta_{k}}\right)\right]$ to obtain multivariate envelope curves ${\left\{e^{\theta_{k}}\,\left(t\right)\right\}}_{k=1}^{K}$.
7. Calculate mean *m*(*t*) of the envelope curves for a set of *K* direction vectors as $m\,\left(t\right)=1/K\times \sum_{k=1}^{K}e^{\theta_{k}}\,\left(t\right)$.
8. Extract “detail” *c*_*i*_(t) using *c*_*i*_(*t*) = *v*(*t*) − *m*(*t*). If the “detail” *c*_*i*_(t) fulfills the stoppage criterion for a multivariate IMF, apply the above procedure to *v*(*t*) − *c*_*i*_(*t*). otherwise, apply it to *c*_*i*_(t).
9. Discard *q* channels corresponding to noise from the resulting (*n* + *q*)-variate IMFs, which gives a set of *n*-channel IMFs corresponding to the original signal.

Herein, steps 3–8 refer to the application of MEMD to the (*n* + *q*)-dimensional multivariate signal.

**FIGURE 3:**
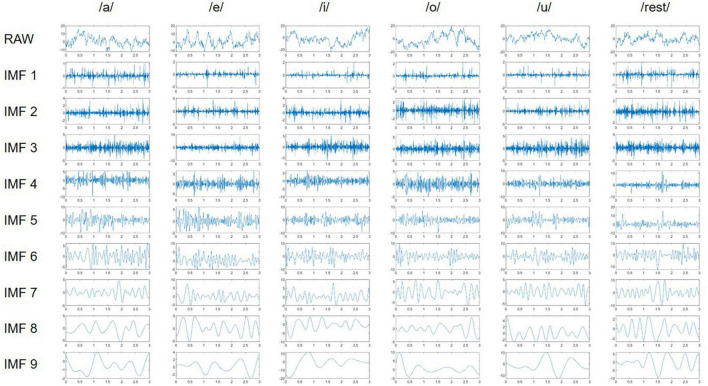
Representation of original EEG (raw) and the extracted IMFs of participant 7 (S7) at channel F5.

As displayed in Figure 3, nine IMFs obtained for each channel using NA-MEMD were calculated from participant 7 (denoted as S7) at channel F5, which covers the Broca’s area (Qureshi et al., 2017). The figure also describes the decomposition dynamics of NA-MEMD for imagined speech EEG. The frequency band of IMF 1 is the highest, while that of IMF 9 is the lowest. Therefore, IMF 1, which has the highest frequency band, was excluded from this study. Six statistical features, namely mean, absolute mean, variance, standard deviation, skewness, and kurtosis, were obtained from each IMF of each channel, and feature vectors were constructed and used as input to the classifier. The mean value and absolute mean are the measurements of the arithmetic average of the signal and absolute signal, respectively (Risqiwati et al., 2020). Standard deviation is a measured amount of variation or signal distribution, and variance is the square of standard deviation (Risqiwati et al., 2020). Skewness is a measure of the asymmetry of the probability distribution of a real-valued random variable, and kurtosis is a measure of the peak or flatness of the probability distribution of a real-valued random variable (Priya et al., 2018).

To confirm the performance of NA-MEMD, WPD was selected as a comparative signal decomposition method. First, to match the number of used sub-band, 3-level WPD with Daubechies 2 wavelet was conducted (Zainuddin et al., 2018). Then, from the eight wavelet coefficients generated by WPD, six statistical features, which were the same as those for the IMF of NA-MEMD, were calculated. Finally, feature vectors were constructed and used as input to the same classifier.

### 2.4. Multireceptive field CNN

Convolutional neural networks are one of the most popular feed-forward neural networks, and their structure is inspired by the human brain’s visual cortex (Dai et al., 2020). They generally comprise convolutional layers, pooling layers, and fully connected layers. The convolutional layer and pooling layer have higher-dimensional features from the input, and the fully connected layer mainly plays the role of classification. CNNs have been used in many bio-signal processing because they mimic the essential characteristics of the inspired cerebral cortex, such as local connectivity, invariance to location, and local transition.

One of the characteristics of CNNs is that the units in individual layers can extract features only from specific samples called receptive fields (RF) (Schirrmeister et al., 2017). A way to further narrow down the features likely to be used is to use domain-specific prior knowledge and investigate whether CNNs have learned known class discrimination functions. After that, they calculate the function values of all RFs extracted by all individual units for each class-distinguishing ability, giving the effect of this function on the unit output.

**FIGURE 4:**
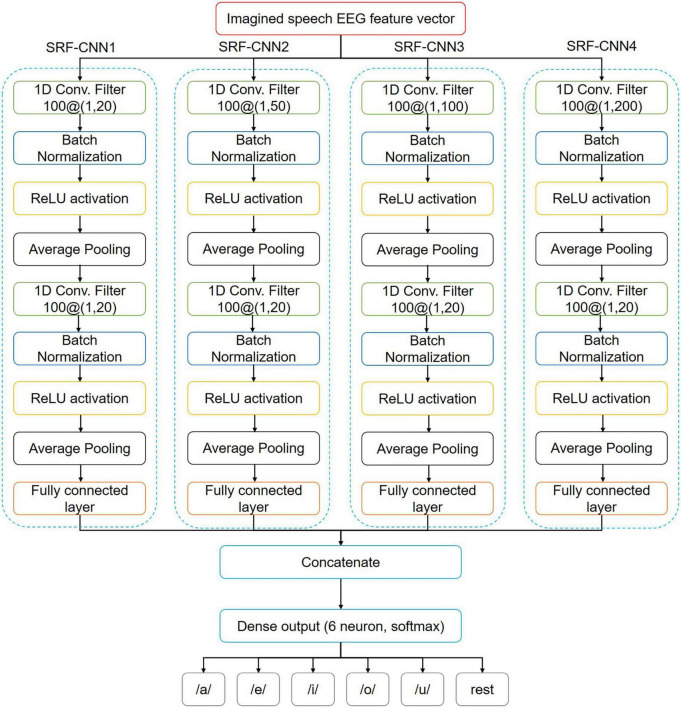
Architecture of the proposed MRF-CNN.

The optimal RF may vary from participant to participant. It may even change for the same participant (Gao et al., 2022). Therefore, we propose the MRF-CNN approach, combining CNNs with multiple RFs, as shown in Figure 4. MRF-CNN consists of four single-receptive field convolutional neural networks (SRF-CNNs). Each SRF-CNN has different kernel sizes at the first convolutional layer, which produces a different size of the RF and can therefore extract broad ranges of high-dimensional features. For instance, SRF-CNN1 comprises the first convolutional layer with a kernel size of 1 × 20, which provides the smallest RF compared to other SRF-CNNs. Each SRF-CNN adds a batch normalization layer after the convolution layer to improve the training process and mitigate overfitting. The ReLU activation function follows an average pooling layer. Moreover, each SRF-CNN consists of two convolutional layers and one fully connected layer. Therefore, the output of the fully connected layer is concatenated and followed by the soft-max layer to classify six imagined speech EEG.

In our study, the initial learning rate and the iteration of the CNN-based classification method were set to 0.001 and 2,000, respectively. The adaptive moment estimation optimizer was used during the training process. Our graphics processor is NVIDIA GeForce RTX 2080 TI with 11 GB RAM, and all computational procedure was done using MATLAB (Mathwork, Inc., USA). To check the performance of deep MRF-CNN, various machine learning methods, SRF-CNN, shallow MRF-CNN, and various end-to-end CNN-based deep learning were used for comparison. The SVM with the linear kernel (*SVM*_*lin*_), SVM with radial basis function kernel (*SVM*_*rbf*_), linear discriminant analysis (LDA), and *k*-nearest neighbor (KNN) method were used for classification in conventional machine learning. The kernel parameters for SVM were calculated as appropriate through a heuristic procedure using subsampling during training. The number of neighbors is chosen to be four which has the best performance in KNN for our data. EEGNet (Lawhern et al., 2018), deepConvNet (Schirrmeister et al., 2017), ShallowConvNet (Schirrmeister et al., 2017), and Channel-wise Convolution with Channel Mixing (C2CM) (Sakhavi et al., 2018) were also used for comparison end-to-end CNN-based deep learning methods. To compare the results statistically, the proposed MRF-CNN method and the results of each classifier were reached through a paired *t*-test to calculate statistical significance.

## 3. Results

### 3.1. Classification results

Table 1 displays the classification results of the proposed method and other methods used for comparison. Herein, S1–S9 refer to participants 1–9, respectively. The average classification accuracy and standard deviation were calculated for each subject using *k*-fold cross-validation. In the *k*-fold cross-validation, all the trials were randomly divided into *k* subsets with the same size. After dividing *k* subsets, one subset was randomly selected to be used as a testing set, and the others were used as a training set of classifiers. In this study, we used 10 × 10-fold cross-validation (*k* = 10), which repetes ten times of 10-fold cross-validation.

**TABLE 1:** Comparison of the average classification accuracy for all participants of the proposed method with machine learning methods and other signal decomposition methods.

	S*VM*_l*in*_	S*VM*_r*bf*_	LDA	KNN	Shallow MRF-CNN	Deep MRF-CNN	Deep MRF-CNN with WPD
S1	61.16 (±4.00)	73.74 (±3.54)	61.42 (±4.24)	68.64 (±5.97)	70.92 (±5.23)	**79.51** (±3.51)	75.25 (±3.65)
S2	44.93 (±4.03)	**64.33** (±4.07)	48.81 (±4.28)	62.12 (±4.42)	46.00 (±16.87)	64.17 (±5.29)	52.39 (±5.49)
S3	52.79 (±4.59)	68.30 (±4.15)	57.85 (±4.92)	65.07 (±4.81)	57.87 (±7.73)	**70.53** (±4.84)	53.46 (±5.91)
S4	53.55 (±3.86)	67.41 (±4.85)	52.30 (±4.61)	62.89 (±4.20)	68.29 (±4.71)	**74.45** (±3.98)	67.74 (±5.04)
S5	67.65 (±3.79)	74.70 (±3.64)	60.20 (±4.03)	67.10 (±4.19)	72.19 (±7.56)	**80.41** (±6.27)	78.65 (±3.49)
S6	52.69 (±4.50)	67.41 (±4.11)	50.60 (±4.40)	67.27 (±4.36)	65.41 (±4.71)	**73.74** (±3.93)	64.84 (±4.89)
S7	55.35 (±3.64)	66.25 (±3.97)	53.52 (±3.90)	61.60 (±4.22)	66.78 (±9.81)	**72.28** (±4.34)	69.40 (±5.16)
S8	58.66 (±4.52)	67.86 (±3.77)	53.83 (±4.24)	63.28 (±4.34)	72.82 (±5.82)	**76.53** (±4.35)	69.34 (±4.20)
S9	50.95 (±4.09)	**67.86** (±4.03)	50.33 (±4.85)	62.89 (±4.20)	56.04 (±3.98)	66.16 (±5.81)	50.19 (±5.03)
Average	55.30	68.65	54.32	64.54	64.04	**73.09**	64.59

The bold values indicate the classification values of the classifier that performed the highest in each subject.

The average classification rate of the proposed method is approximately 73%, which is higher than the classification accuracy of the other methods with different CNN architectures. In addition, the results of the proposed MRF-CNN show statistically significant differences (*p*-value < 0.05) from those of other classifiers via paired *t*-test.

We compared the performance of NA-MEMD with WPD to evaluate the performance of NA-MEMD. First, a three-level WPD was used to match the number of sub-bands used. Next, the same six statistical features were used using eight wavelet coefficients. As per Table 1, NA-MEMD and the proposed MRF-CNN outperform WPD with MRF-CNN, which means the signal decomposition performance of NA-MEMD is better than WPD.

Table 2 displays the results of SRF-CNN and the proposed MRF-CNN. The proposed MRF-CNN shows a statistically significant difference (*p*-value < 0.05) from the results of each deep SRF-CNN via paired *t*-test. This is because each SRF-CNN analyzes only a specific region of the input feature vector, whereas MRF-CNN covers and analyzes small to large ranges.

**TABLE 2:** Average classification accuracy for all participants in MRF-CNN and SRF-CNN.

Participant	Deep MRF-CNN	Deep SRF-CNN1	Deep SRF-CNN2	Deep SRF-CNN3	Deep SRF-CNN4
S1	**79.51** (±3.51)	73.90 (±5.34)	75.15 (±6.51)	74.16 (±7.16)	75.39 (±7.07)
S2	**64.17** (±5.29)	50.21 (±9.52)	51.06 (±13.68)	48.57 (±15.36)	50.09 (±13.62)
S3	**70.53** (±4.84)	58.31 (±9.67)	58.44 (±11.66)	57.03 (±13.71)	55.12 (±15.14)
S4	**74.45** (±3.98)	67.16 (±8.98)	63.64 (±11.97)	68.77 (±6.32)	65.39 (±13.89)
S5	**80.41** (±6.27)	79.51 (±6.40)	76.57 (±8.11)	76.98 (±9.91)	74.37 (±14.00)
S6	**73.74** (±3.93)	62.57 (±14.44)	63.81 (±13.05)	63.36 (±14.44)	62.81 (±9.65)
S7	**72.28** (±4.34)	66.80 (±9.39)	67.63 (±7.65)	66.95 (±8.96)	67.65 (±10.77)
S8	**76.53** (±4.35)	69.52 (±11.87)	70.36 (±9.11)	70.09 (±9.82)	67.97 (±13.39)
S9	**66.16** (±5.81)	52.20 (±9.87)	49.12 (±13.35)	47.26 (±14.26)	43.65 (±15.59)
Average	**73.09**	64.46	63.98	63.69	62.49

The bold values indicate the classification values of the classifier that performed the highest in each subject.

**FIGURE 5:**
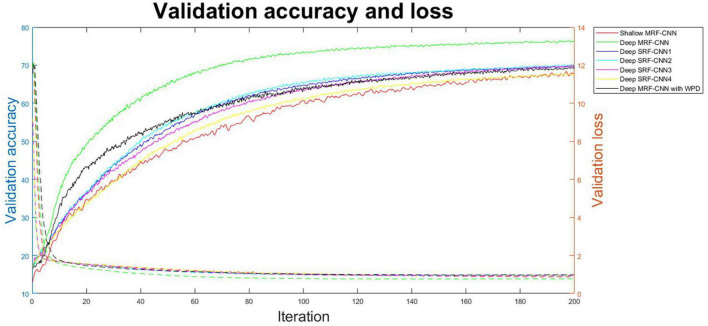
Average validation accuracy and loss of each iteration during training at participant 8 (S8).

The proposed method’s validation classification accuracy and loss were compared with several CNNs to evaluate its robustness. Figure 5 displays each iteration’s validation classification rate and loss for S8. As per the figure, the proposed method exhibits the most effective classification accuracy and loss in the least iteration. Therefore, the proposed MRF-CNN method is more efficient in classifying imagined speech EEG than other neural networks.

Table 3 shows the results of the proposed MRF-CNN framework and various end-to-end CNN-based deep learning methods. Unlike the proposed framework, end-to-end CNN-based deep learning methods use raw EEG signals as input. Table 3 demonstrates that using features as input for deep learning, like the proposed method, is better than using raw EEG as input for deep learning. Also, the proposed method showed a statistically significant difference (*p*-value < 0.05) from other end-to-end CNN-based deep learning methods.

**TABLE 3:** Comparison of the average classification accuracy for all participants of the proposed method with other end-to-end deep learning methods.

Participant	Deep MRF-CNN	EEGNet 8,2 (Lawhern et al., 2018)	DeepConvNet (Schirrmeister et al., 2017)	ShallowConvNet (Schirrmeister et al., 2017)	C2CM (Sakhavi et al., 2018)	Deep MRF-CNN with WPD
S1	**79.51** (±3.51)	47.31 (±2.84)	58.23 (±3.27)	45.31 (±4.38)	57.48 (±4.89)	75.25 (±3.65)
S2	**64.17** (±5.29)	33.72 (±4.80)	35.84 (±5.32)	28.34 (±3.39)	36.18 (±5.25)	52.39 (±5.49)
S3	**70.53** (±4.84)	37.46 (±1.97)	40.00 (±5.18)	22.97 (±3.93)	35.09 (±5.40)	53.46 (±5.91)
S4	**74.45** (±3.98)	50.53 (±7.37)	56.20 (±7.46)	48.39 (±4.67)	47.81 (±4.45)	67.74 (±5.04)
S5	**80.41** (±6.27)	77.31 (±2.44)	79.93 (±5.86)	63.30 (±4.47)	72.13 (±3.14)	78.65 (±3.49)
S6	**73.74** (±3.93)	39.14 (±6.73)	49.42 (±3.42)	40.58 (±2.92)	45.61 (±3.63)	64.84 (±4.89)
S7	**72.28** (±4.34)	51.38 (±6.91)	47.77 (±4.56)	43.62 (±4.54)	45.85 (±3.50)	69.40 (±5.16)
S8	**76.53** (±4.35)	53.20 (±6.87)	57.60 (±4.76)	47.42 (±5.53)	46.57 (±3.42)	69.34 (±4.20)
S9	**66.16** (±5.81)	33.75 (±4.06)	38.21 (±4.19)	29.91 (±2.74)	40.14 (±4.50)	50.19 (±5.03)
Average	**73.09**	47.09	51.47	41.09	47.43	64.59

The bold values indicate the classification values of the classifier that performed the highest in each subject.

As per the classification results, the classification accuracy is higher than the chance level in all classifiers, indicating the effectiveness of imagined speech EEG. Therefore, vowel imagery EEG can be used as the ultimate substitute as an alternative task for other BCIs.

### 3.2. Frequency sub-band analysis

Table 4 presents the proposed CNN classification accuracy for each IMF of NA-MEMD. Similar to the results of previous studies (Min et al., 2016; Kaongoen et al., 2021), the speech imagery information is primarily contained around the higher frequency band regions (IMF 2 to IMF 4) and in the lowest frequency band region (IMF 9) of imagined speech EEG. However, since the classification accuracy exceeds the chance level in all other frequency bands, those bands also seem to have information for classifying speech imagery EEG. Therefore, in Table 4, the higher frequency band region (IMF 2 to IMF 4) may contain more information to help classify imagined speech EEG than other lower frequency bands besides IMF 9.

**TABLE 4:** Average classification accuracy for all participants in each IMFs using MRF-CNN.

	IMF 2	IMF 3	IMF 4	IMF 5	IMF 6	IMF 7	IMF 8	IMF 9	All
S1	65.39 ( ±5.87)	73.65 ( ±4.23)	67.09 ( ±5.14)	53.87 ( ±3.04)	40.14 ( ±5.72)	40.89 ( ±2.93)	44.94 ( ±4.45)	59.56 ( 5.00)	**79.51** ( ±3.51)
S2	50.95 ( ±4.55)	60.14 ( ±4.45)	48.13 ( ±4.77)	44.80 ( ±4.78)	40.28 ( ±2.80)	37.46 ( ±4.64)	38.31 ( ±3.34)	54.27 ( ±3.47)	**64.17** ( ±5.29)
S3	49.41 ( ±4.17)	57.03 ( ±4.73)	47.71 ( ±5.83)	42.20 ( ±5.86)	37.46 ( ±4.35)	41.69 ( ±4.53)	44.83 ( ±7.63)	63.39 ( ±5.98)	**70.53** ( ±4.84)
S4	58.00 ( ±4.41)	66.96 ( ±4.17)	58.63 ( ±4.39)	42.80 ( ±4.05)	41.21 ( ±3.99)	40.36 ( ±3.69)	44.80 ( ±3.88)	59.85 ( ±4.93)	**74.45** ( ±3.98)
S5	63.77 ( ±6.17)	73.94 ( ±4.74)	64.85 ( ±4.94)	44.37 ( ±7.11)	36.98 ( ±5.39)	34.48 ( ±3.52)	41.82 ( ±4.21)	53.40 ( ±3.86)	**80.41** ( ±6.27)
S6	58.56 ( ±5.34)	65.68 ( ±4.41)	56.83 ( ±4.49)	43.31 ( ±4.00)	39.57 ( ±3.75)	42.16 ( ±2.86)	40.94 ( ±4.72)	55.61 ( ±5.04)	**73.74** ( ±3.93)
S7	63.31 ( ±3.03)	70.62 ( ±3.20)	59.38 ( ±5.49)	44.46 ( ±4.23)	34.69 ( ±3.33)	39.23 ( ±4.85)	39.23 ( ±3.73)	48.38 ( ±6.07)	**72.28** ( ±4.34)
S8	68.41 ( ±3.95)	67.21 ( ±3.74)	61.69 ( ±4.49)	45.09 ( ±4.21)	42.06 ( ±3.33)	39.44 ( ±4.32)	42.26 ( ±3.28)	56.90 ( ±3.25)	**76.53** ( ±4.35)
S9	48.32 ( ±5.52)	51.34 ( ±2.89)	47.57 ( ±5.09)	43.29 ( ±5.51)	38.63 ( ±4.90)	38.83 ( ±3.33)	41.58 ( ±3.61)	55.88 ( ±4.08)	**66.16** ( ±5.81)
Average	58.46	65.17	56.88	44.91	39.00	39.39	42.08	56.36	**73.09**

The bold values indicate the classification values of the classifier that performed the highest in each subject.

### 3.3. Confusion matrix

**FIGURE 6:**
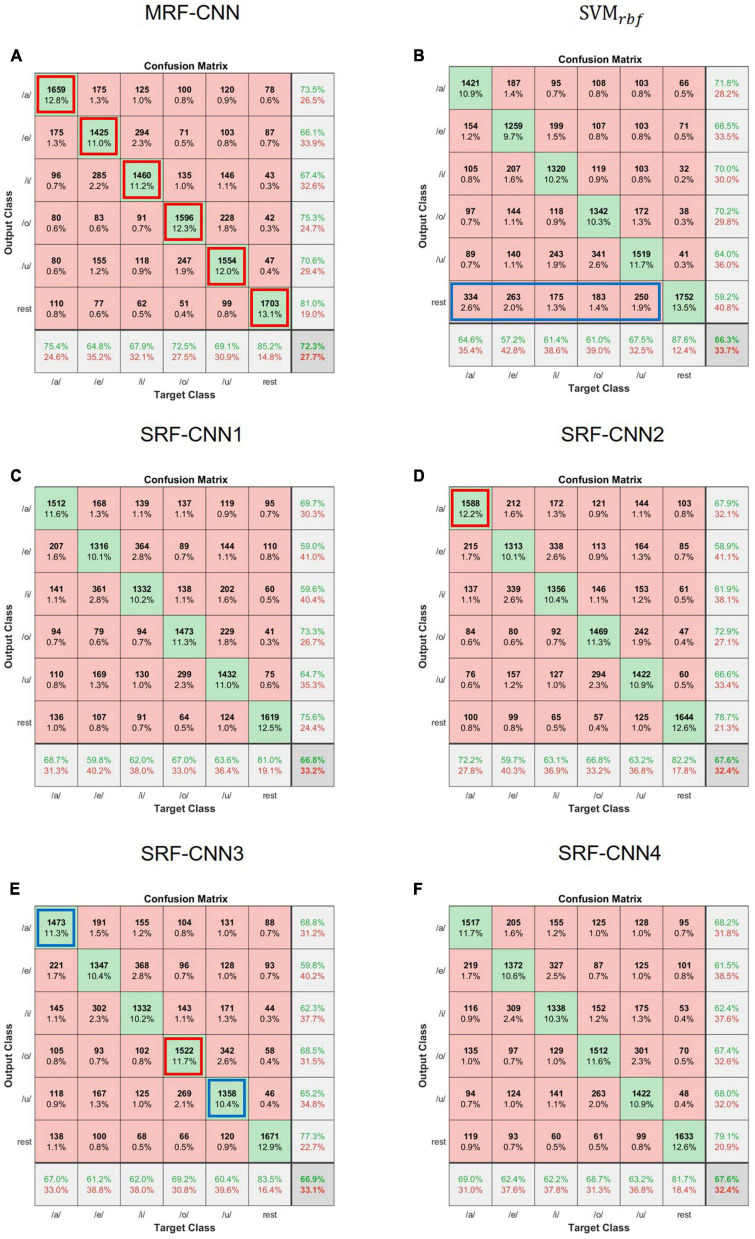
Confusion matrix of **(A)** MRF-CNN, **(B)** *SVM*_*rbf*_, **(C)** SRF-CNN1, **(D)** SRF-CNN2, **(E)** SRF-CNN3, and **(F)** SRF-CNN4 of participant 7 (S7).

We used a confusion matrix to analyze the sensitivity of each class to determine which imagined vowel affects the classification accuracy of classifiers. Figure 6 represents the results of the confusion matrix of MRF-CNN, four SRF-CNNs, and SVM with radial basis function kernel of S7. As per the figure, the probability of misclassifying imagined vowel /e/ as /i/ or vice versa is higher than other misclassifications in the deep learning approach. In addition, the probability of misclassifying the imagined vowel /o/ as /u/ or vice versa is higher. Next, MRF-CNN and other classifiers classify resting-state EEG well. However, unlike SVM, MRF-CNN does not classify imagined vowels as resting-state EEG, as seen in the blue box in Figure 6B. Comparing each SRF-CNN classifier reveals advantages and disadvantages in classifying one or two imagined vowel EEG in SRF-CNN, as seen in the red boxes and blue boxes in Figures 6D, E. For instance, SRF-CNN2 classifies the imagined vowel /a/ more accurately than other SRF-CNNs. However, MRF-CNN exhibits higher classification accuracy than all SRF-CNNs.

## 4. Discussion

In this study, we proposed NA-MEMD and MRF-CNN for imagined speech EEG-based BCIs.

As deep learning has been developed and improved, it has been applied to various fields and is widely used in EEG (Altaheri et al., 2021; Rahman et al., 2021; Aggarwal and Chugh, 2022). In several previous studies, MRF-CNN performed better classification of multiple images and signal data than other SRF-CNNs, as shown by analyzing numerous datasets (Hu et al., 2019; Dai et al., 2020; Liu et al., 2020). However, no other studies have used NA-MEMD and MRF-CNN for imagined speech EEG signals.

A previous end-to-end deep learning paper mentioned that the deeper layer could extract more global and high-level features (Schirrmeister et al., 2017). For example, the deeper layer can detect complex visual features such as edges, shapes, and objects from raw images in image processing. In this study, statistical features of the decomposed EEG were extracted and used as input to the proposed deep learning architecture. Tables 1, 3 show that the proposed method outperforms other signal decomposition and classification methods. Table 3 shows that the proposed method outperforms other end-to-end CNN-based deep learning. It means the high-level features from statistical features have more discriminative information to classify imagined speech EEG than raw EEG. Also, Tables 1, 3 demonstrate that machine learning outperforms end-to-end CNN-based deep learning. It means that the high-level features from raw EEG did not have as much discriminative information for imagined speech EEG as the statistical features of the EEG. Therefore, statistical features are more effective than simply giving raw EEG as input.

Unlike the MI BCI, which is known to be mainly focused on the alpha and beta bands, in the imagined speech EEG-based BCI, research on which frequency band is related to imagined speech EEG is being actively conducted (Zhu et al., 2019; Altaheri et al., 2021; Kaongoen et al., 2021; Mini et al., 2021; Lopez-Bernal et al., 2022). In this study, the proposed method provided more promising results in the gamma and delta bands than in other bands. However, since it exhibited a much higher classification rate than the chance level in all bands, we concluded that the information related to speech imagination is included across all brain wave bands. Some previous papers have reported that most information about speech imagination is in the delta, beta, and gamma bands of speech imagination EEG (Kaongoen et al., 2021; Mini et al., 2021). Furthermore, it has been reported that other bands contain less information related to speech imagination (Tripathi, 2022). However, when we used NA-MEMD to decompose brain waves, we found that a certain amount of information related to speech imagination exists across all bands and that the gamma and delta bands have the most information. Furthermore, several ECoG studies have reported that the high gamma band contains information related to speech processing (Greenlee et al., 2011; Llorens et al., 2011; Martin et al., 2016; Panachakel and Ramakrishnan, 2021a). Therefore, the results of this study showed that EEG has information related to speech imagination in delta regions and gamma regions, which is consistent with the results presented in previous imagined speech processing papers.

Currently, many communication systems that apply various BCI technologies are being developed for commercialization. For example, the P300 speller has been most widely studied for decoding spoken thoughts from EEG but requires long concentration time and high processing times for good performance. Another example is MI BCI, which requires a short concentration and low processing time to get results. However, it can only decipher a limited number of imagined movements. Imagined speech-based BCI can overcome all of the abovementioned drawbacks, which has resulted in many research studies. However, few studies can multi-classify imaginary speech EEG, and their results are too difficult to commercialize. This study improved the imagined speech EEG-based BCIs in terms of multi-class classification accuracy, and further studies will be conducted to develop a practical and generalized BCI system.

## 5. Conclusion

The primary purpose of this study was to test classification performances for imagined speech EEG using signal decomposition methods and deep CNNs. The study results concluded that combining NA-MEMD as the signal decomposition for EEG and MRF-CNN is the most effective method for classifying imagined speech EEG. Furthermore, this study demonstrated statistically significant differences (*p*-value < 0.05) between the proposed method and other signal decomposition and classification methods. However, the proposed method has limitations in real-time applications due to too much preprocessing time. Also, it has not been applied to other BCI applications. In a future analysis, we will advance the proposed approach for other BCI application.

## Data availability statement

The raw data supporting the conclusions of this article will be made available by the authors, without undue reservation.

## Ethics statement

The studies involving human participants were reviewed and approved by the International Review Board of Gwangju Institute of Science and Technology. The patients/participants provided their written informed consent to participate in this study.

## Author contributions

HP designed the experiment protocol and implemented the methodologies, and drafted the manuscript. BL supervised the entire research process and revised the manuscript. Both authors contributed to the results interpretation and proofreading of the manuscript.
